# Seronegative Paraneoplastic Limbic Encephalitis in Non-Small Cell Lung Cancer: A Case of Neuropsychiatric Symptoms Resolving After Resection

**DOI:** 10.1016/j.atssr.2025.11.025

**Published:** 2025-12-19

**Authors:** Neelesh Bagrodia, Peter J. Kneuertz, Desmond M. D’Souza, Robert E. Merritt, Dwight H. Owen, Ioana Baiu

**Affiliations:** 1 Division of Thoracic Surgery, Department of Surgery, The Ohio State University Wexner Medical Center, Columbus, Ohio; 2 Division of Medical Oncology, Department of Medicine, The Ohio State University Wexner Medical Center, Columbus, Ohio

## Abstract

Paraneoplastic syndromes (PNS) are rare manifestations of particular malignancies. PNS symptoms have been postulated to be caused by hormonal secretion or immune cross-reactivity with the tumor. Although classically associated with small cell carcinoma, PNS have been reported across many cancers, including non-small cell carcinoma. We present an unusual case of a patient whose diagnosis of non-small cell carcinoma was discovered through a workup for new onset of neuropsychiatric symptoms that resolved within days after the resection of her primary cancer. Surgical resection of the underlying malignancy remains the fastest and most definitive treatment for paraneoplastic symptoms.

Paraneoplastic syndromes (PNS) represent a broad range of pathologies that can present in patients with certain malignancies. They typically cause a host of secondary effects on the neurologic, endocrine, and rheumatologic systems.^1^ PNS develops in ∼10% to 15% of patients with cancer, and these symptoms often precede the diagnosis of malignancy.^2^ One proposed mechanism for PNS is immune dysregulation incited by the tumor.^2^ For example, the most common PNS associated with lung cancer is the syndrome of inappropriate antidiuretic hormone secretion that presents with unexplained hyponatremia.^1^ Neuropsychiatric symptoms are not classically associated with non-small cell lung cancer (NSCLC) but can be seen in small cell cancers.^3^ We describe a patient who presented with new-onset encephalopathy that manifested primarily as dramatic personality changes, whose work-up revealed a new diagnosis of NSCLC, and symptoms resolved entirely after resection.

A 79-year-old female patient, former smoker with <20 pack-year history, presented with a 2-week history of encephalopathy, manifesting with delirium, visual hallucinations, and social disinhibition—notably with violent verbal outbursts and profanities. She required hospitalization along with a 24/7 sitter and nighttime soft restraints. Of note, she had a history of Guillain-Barré syndrome and the variant Bickerstaff brainstem encephalitis (BBE) that presented 10 and 14 years prior. BBE is a rare autoimmune condition that presents as ataxia, cognitive decline, and ophthalmoplegia, the last of which our patient did not have. Before this, she was a highly functional, active, and independent woman living with her husband.

An extensive neurologic and psychiatric workup over the course of 4 weeks of hospitalization, including brain magnetic resonance imaging, lumbar puncture, and whole-body scans, revealed only a 2.7-cm left upper lobe lung nodule, the biopsy specimen of which was consistent with lung adenocarcinoma, a positive station 11L lymph node, and a 4-mm right upper lobe lung nodule (Figure 1). The results of an autoimmune encephalopathy panel of serum and cerebrospinal fluid were negative. The panel included α-amino-3-hydroxy-5-methyl-4-isoxazolepropionic acid receptor (AMPA-R) antibody; amphiphysin antibody; anti-glial/neuronal nuclear antibody-type 1 (AGNA-1); antineuronal nuclear antibody-types 1, 2, and 3 (ANNA-1 [Anti-Hu], ANNA-2; ANNA-3); contactin-associated protein-2– immunoglobuln G (CASPR2-IgG); collapsin response-mediator protein-5 (CRMP-5) IgG; dipeptidyl-peptidase–like protein 6 (DPPX) antibody; γ-amino butyric acid receptor, type B (GABA-B-R) antibody; glutamic acid decarboxylase 65 (GAD65) antibody; glial fibrillary acidic protein (GFAP); metabotropic glutamate receptor 1 (mGluR1) antibody; IgLON5; leucine-rich, glioma-inactivated 1 (LGI1)-IgG; neurochondrin; neuronal intermediate filament (NIF); *N*-methyl-d-aspartate receptor (NMDAR) antibody; Purkinje cell antibody (PCA) types 1, 2, and TR; phosphodiesterase 10A (PDE10A) IgG; septin-7; and tripartite motif-containing protein 46 (TRIM46) antibody

**Figure 1:**
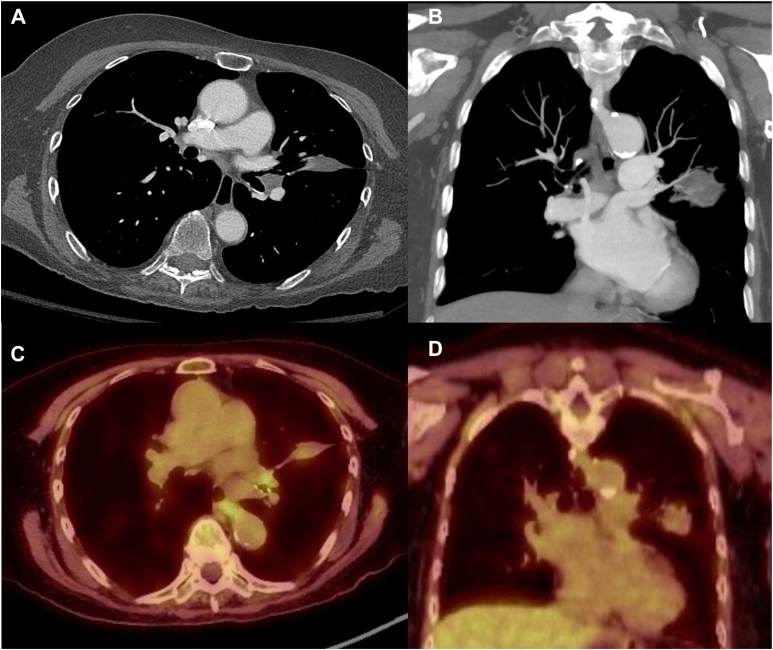
(A, C) Transverse and (B, D) coronal cross-sections on computed tomography and positron emission tomography imaging, respectively, demonstrate the patient’s left-sided non-small cell lung cancer.

The presumptive diagnosis was seronegative PNS, and she was initiated on empiric therapy with high-dose solumedrol and intravenous immunoglobulin without resolution of her symptoms. Small cell lung cancer (SCLC) has been linked to limbic encephalitis, but the association with NSCLC has not been described.^4^ Given N1 disease, the role of neoadjuvant therapy or definitive chemoradiation were discussed in tumor board, but the patient’s mental status was so debilitating that she would not have been able to engage in any type of medical therapy. The decision therefore was to proceed directly to the operating room.

She underwent a single-anesthesia event with navigational bronchoscopy biopsy of the right upper lobe lung nodule, which ruled-out both stage IV disease and concurrent contralateral SCLC, followed by a robotic-assisted left upper lobectomy (Figures 2B, 3). On the evening of postoperative day 0, the patient notably did not require restraints as she had for the preceding weeks. On postoperative day 1, the patient no longer required a sitter because she was calm and compliant with all treatments. In the days after the operation, the patient’s mental status improved until she returned to a very pleasant normal baseline, confirmed by her husband. Final pathology demonstrated T2a N1 M0 NSCLC, adenocarcinoma histology with classical activating *EGFR* L858R mutation as well as mutations in *ERBB2* and *CDKN2A*.

**Figure 2:**
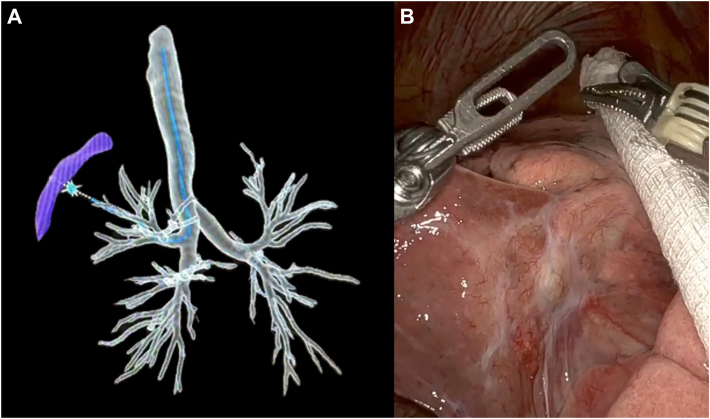
Intraoperative (A) navigational bronchoscopy imaging of the right-sided contralateral nodule and (B) robot-assisted thoracic surgery view of the left-sided lung adenocarcinoma.

**Figure 3:**
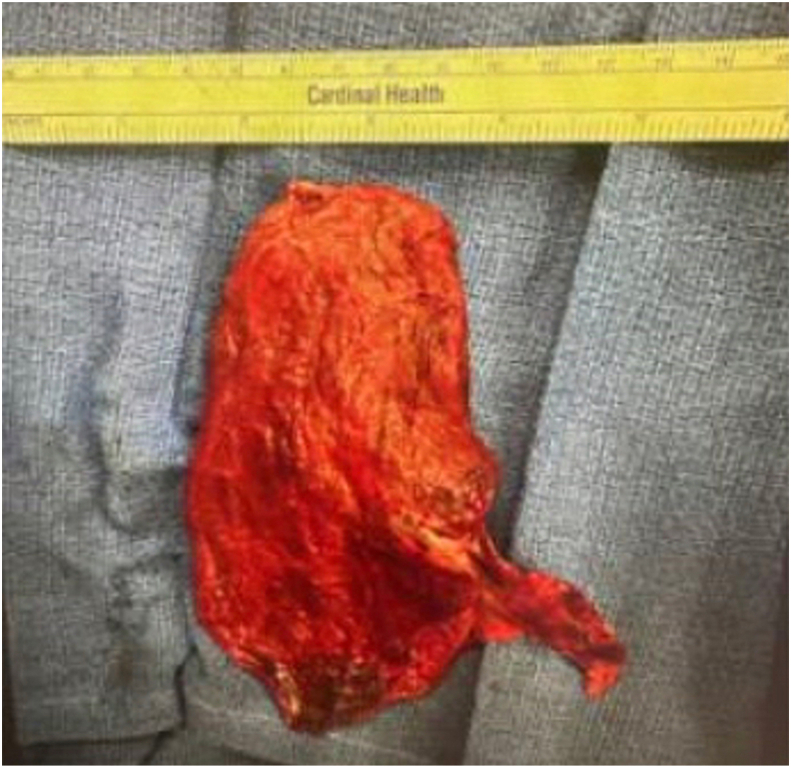
Intraoperative gross specimen appearance.

She was initiated on osimertinib therapy after making a full recovery from surgery based on results of ADAURA (AZD9291 Versus Placebo in Patients With Stage IB-IIIA Non-small Cell Lung Carcinoma, Following Complete Tumour Resection With or Without Adjuvant Chemotherapy).^5^ She remains disease free and her mental status is within normal limits at 6 months of follow-up.

## Comment

This case highlights a unique presentation of NSCLC PNS presenting as a profound encephalopathy and violent outbursts of profanity-laden language. A thorough workup by the neuroimmunology and psychiatry teams revealed only the 2 lung nodules, one of which was not malignant on the biopsy specimen and the other that was a primary lung adenocarcinoma. As such, the diagnosis of seronegative paraneoplastic limbic encephalopathy was made. The resolution of neurologic symptoms in temporal relationship to the surgical excision confirms PNS as the most likely etiology.

Lung cancer is known to cause PNS in 10% to 20% of patients.^1^ Squamous cell carcinoma of the lung can result in humoral hypercalcemia of malignancy, whereas syndrome of inappropriate antidiuretic hormone secretion, ectopic Cushing syndrome, or Lambert-Eaton myasthenia syndrome develops in 40% of patients with SCLC.^1^^,^^4^ It is important to note that whereas PNS are related to the underlying malignancy, they are not a sign of metastasis.^1^ An autoimmune-like uncontrolled release of humoral mediators, such as hormones, cytokines, and antibodies, has been proposed as a mechanism.^4^ Our patient did have a history of Guillain-Barré syndrome and BBE, and therefore, a predisposition to autoimmune-like phenomena. In an analysis of PNS among patients with NSCLC, anti-Hu and anti–γ-aminobutyric and antibodies were prevalent in 40% and 33% of serum samples, respectively.^3^ However, the lack of complete penetrance suggests that there are multifactorial underlying mechanisms to the presentation of PNS.

The acute changes in personality and use of vulgar language, as seen in our patient, have been observed in patients with autoimmune encephalitis (AE).^6^ AE frequently presents with psychiatric symptoms and is strongly linked to anti–NMDAR antibodies.^6^ A previous case identified SCLC as the etiology for NMDAR antibodies-associated AE.^3^^,^^7^ Given the negative AE panel workup in our patient and her lack of response to empiric steroid therapy before surgical resection, what the neurochemical basis was for her symptoms remains unknown. Nonetheless, her postoperative course and rapid recovery to a normal baseline underscores that treatment of the underlying malignancy provides optimal management of PNS. Even when the association between the malignancy and the PNS is still in question, surgical resection can achieve the fastest and most definitive resolution of symptoms in patients and ultimately confirm the diagnosis.
